# Anti-inflammatory Activity of Hautriwaic Acid Isolated from *Dodonaea viscosa* Leaves

**DOI:** 10.3390/molecules17044292

**Published:** 2012-04-10

**Authors:** David Osvaldo Salinas-Sánchez, Maribel Herrera-Ruiz, Salud Pérez, Enrique Jiménez-Ferrer, Alejandro Zamilpa

**Affiliations:** 1 Biodiversity and Conservation Research Center. (UAEM), Av. Universidad 1001, Col. Chamilpa, Cuernavaca 62209, Morelos, Mexico; Email: osval1671@hotmail.com; 2 Biomedical Research Center of the South (IMSS), Argentina 1, Col. Centro, Xochitepec 62790, Morelos, Mexico; Email: edanae10@yahoo.com.mx (M.H.-R.); enriqueferrer_mx@yahoo.com (E.J.-F.); 3 Department of Biological Systems, (UAM-Xochimilco), Calzada del Hueso 1100, Col. Villa Quietud, México D.F. 04960, Mexico; Email: msperez46@yahoo.com.mx

**Keywords:** anti-inflammatory activity, *Dodonaea viscosa*, hautriwaic acid, TPA

## Abstract

The aim of this study was to identify an anti-inflammatory compound from *D. viscosa *leaves. The structure of this bioactive substance was elucidated by IR and NMR studies, which indicated that this natural product corresponds to hautriwaic acid (HA). This diterpene exhibited good anti-inflammatory activity in 12-*O*-tetradecanoylphorbol 13-acetate (TPA) mice ear edema models by applications at doses of 0.25, 0.5 and 1.0 mg/ear (60.2, 70.2 and 87.1% inhibition, respectively); additionally *Dodonaea viscosa* dichloro-methane extract (DvDE) displays a 97.8% anti-inflammatory effect at 3 mg/kg. Multiple applications of DvDE at doses of 100 mg/kg on TPA mice ear edema inhibited the edema-associated inflammation by 71.8%, while HA at doses of 15 mg/kg, reduced edema to 64% and indomethacin 40%.

## 1. Introduction

Inflammation is implicated in several chronic-degenerative diseases like cancer, diabetes and hypertension. These health problems cause high mortality and morbidity levels around the world. Other inflammation-related diseases like infections by bacteria, virus and protozoa or autoimmune diseases like arthritis or Alzheimer’s are relevant. The main treatments used to prevent or minimize the progression of inflammation include non-steroidal anti-inflammatory drugs (NSAIDs) and corticosteroids, but they are known to have deleterious side effects [1]. Traditional medicine has been used to address human health demands and nowadays it could be considered as the most important source of new anti-inflammatory compounds [2]. The overexposure to the irritant substance 12-*O*-tetradecanoylphorbol 13-acetate (TPA) induces oxidative stress, cutaneous inflammation, and epidermal hyperplasia because it increases the proliferation of keratinocytes and the production of the pro-inflammatory cytokine tumor necrosis factor-alpha (TNF-α) and the formation of leukotrienes (LTB4), which result in an increase of vascular permeability and neutrophil influx [3]. Chronic TPA application (five times in 10 days) in mice induces a prolonged inflammatory reaction characterized by the increase in mice ear thickness, infiltration of inflammatory cells (polymorphonuclear leukocytes), and epidermal hyperplasia [4]. This toxic compound induces the release of interleukin (IL)-1α from the keratinocytes and also from the *de novo *genetic expression of cytokines [5]. Some anti-inflammatory drugs are the first choice in treatment of this last medical disorder. *Dodonaea viscosa* is widely used by local healers in the Reserva de la Biosfera Sierra de Huatla (REBIOSH), located in Morelos, Mexico, for the treatment of inflammation, pain, and rheumatism [6]. Several pharmacological studies have demonstrated the anti-diarrheal, antibacterial, analgesic, antiviral, antiulcer, antioxidant and anti-inflammatory activities of this medicinal plant [7,8,9,10,11,12]. Previous phytochemical studies indicated that *D. viscosa* contains flavonoid- and terpenoid-type secondary metabolites [13,14,15,16]. The objective of the present work was to obtain and identify the most abundant terpene from *D. viscosa* and evaluate its anti-inflammatory properties in both chronic and acute *in vivo* inflammation models.

## 2. Results and Discussion

**Figure 1:**
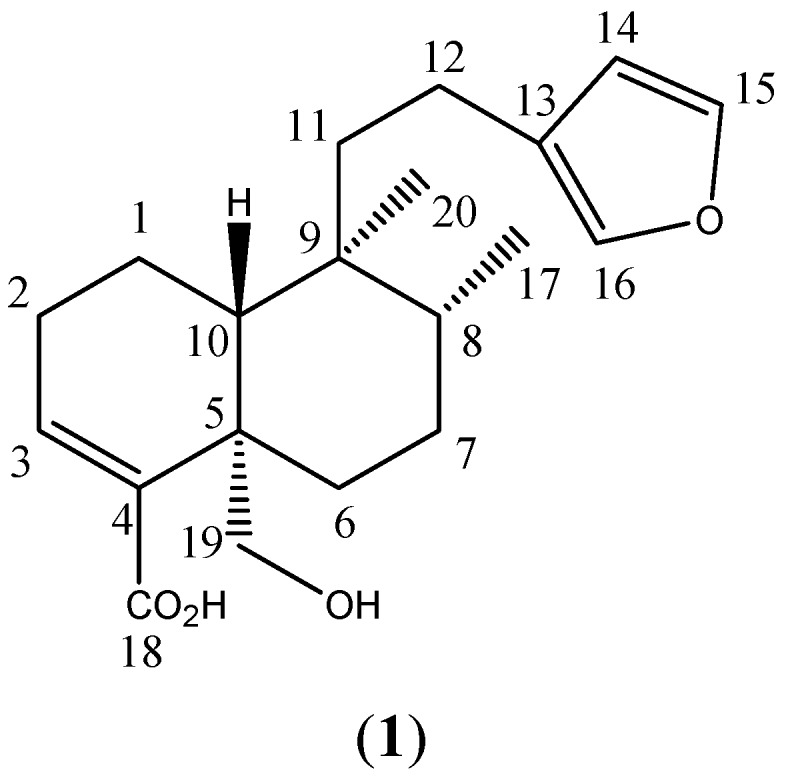
Hautriwaic acid.

DvDE displayed an important anti-inflammatory activity. Open column chromatographic purification of this extract allowed us to obtain an anti-inflammatory terpenoid mixture (F1). Recrystallization of this fraction produced a colorless solid compound that displayed potentanti-inflammatory effects. This purified compound was identified as hautriwaic acid (1, Figure 1) by analysis of its ^1^H- and ^13^C-NMR and infrared spectroscopy data as it showed similar ^1^H- and ^13^C-NMR signals (summarized in the Experimental section) to this *ent*-clerodane diterpene, which was previously isolated from this plant [17,18]. Characteristic signals for HA at 2,921, 2,848, 1,652, 1,447 and 870 cm^−1^ were observed in the IR spectrum.

### 2.1. Anti-Inflammatory Activity

The anti-inflammatory effect of the dichlorometane extract was tested at doses of 3 mg/ear on a TPA-induced edema model, where it displayed a significant inhibition of 97.8% of the edema (Table 1). 

**Table 1:** Anti-inflammatory activity of dichloromethane extract (DvDE), Fraction 1 (F1) and indomethacin (Indo) on TPA induced edema model in CD-1 mice.

Substance	Time (h)	Dose (mg/ear)	Edema (mg) mean ± SEM	Edema inhibition (%)
TPA	6	---	9.88 ± 0.64	---
DvDE	6	3	0.21 ± 0.09 *	97.8 ± 0.9
F1	6	1	0.3 ± 0.10 *	96.9 ± 1.1
Indo	6	1	0.71 ± 0.43 *	92.7 ± 6.2

* *p* < 0.05 in comparison with TPA group.

HA was tested at doses of 0.25, 0.5, and 1.0 mg/ear on TPA-induced inflammation in CD-1 mouse auricular pavilion and showed percentages of inhibition of inflammation of 60.2, 70.2, and 87.1% respectively. At doses of 1.0 mg/ear the activity was similar to that of the positive control indomethacin (86%, 1 mg/ear, Table 2). 

**Table 2:** Ascendant doses evaluation of HA in TPA-induced inflammation model.

Substance	Time (h)	Dose (mg/ear)	Edema (mg) mean ± SEM	Edema inhibition (%)
TPA	6	---	9.8 ± 0.9	---
HA	6	0.25	3.9 ± 0.6 *	60.2 ± 6.5
HA	6	0.5	2.9 ± 0.7 *	70.2 ± 7.4
HA	6	1.0	1.2 ± 0.4 *	87.1 ± 4.4
Indo	6	1.0	1.3 ± 0.8 *	86.0 ± 6.4

* *p* < 0.05 in comparison with TPA group; HA showed an E max = 97% of edema inhibition with an ED_50_ = 0.158 mg/ear.

Bonferroni post-test indicates that both indomethacin results (Table 1 and Table 2) were not significantly different. The anti-inflammatory activity of DvDE (100 mg/kg) and HA (15 mg/kg) administered via the intraperitoneal route, was demonstrated in a chronic TPA assay. These treatments showed an important inhibition of edema 71.8% (DvDE) and 64% (HA), respectively. Indometacin (5 mg/Kg) displayed an edema inhibition of 40% (Table 3). 

Some medicinal species with anti-inflammatory activity produce clerodane diterpenes and furanoclerodanes. *Baccharis incarum* produced a marked anti-inflammatory action in the carrageenan foot edema test, and contains a neoclerodane diterpenoid named bincatriol [19]. Other neoclerodane diterpenes that exert potent anti-inflammatory effect are *E*-isolinaridial and its methyl ketone derivative from *Linaria saxatilis* [20]. Although hautriwaic acid and other furoclerodane diterpenoids have been isolated from anti-inflammatory species [21,22], this is the first time that this biological effect has been shown for hautriwaic acid. Further studies examining the activity of *D. viscosa* in rheumatoid arthritis and other desease-specific models of inflammation are warranted. Because of that it is pertinent to propose *Dodonaea viscosa *for testing in rheumatoid arthritis (RA) models.

**Table 3:** Percentage of inhibition (%) of the DvDE, HA and indomethacin (Indo).

Substance	Time (day-h)	Dose (mg/Kg)	Edema (mg) mean ± SEM	Edema inhibition (%)
TPA	10-6	---	13 ± 2.6	---
DvDE	10-6	100	3.8 ± 1.06 *	71.8 ± 8.4
HA	10-6	15	4.6 ± 1.03 *	64 ± 7.9
Indo	10-6	5	7.8 ± 0.85 *	40 ± 6.5

All treatments were administrated by intraperitoneal (*i.p.*) via. CD-1 mice were used in this topical TPA induced inflammation test. * *p* < 0.05 in comparison with TPA group.

## 3. Experimental

### 3.1. General

12-*O*-Tetradecanoylphorbol 13-acetate (TPA) and indomethacin (Indo) were purchased from Sigma Chemical Co. (St. Louis, MO, USA). Dichloromethane, acetone, and ethyl acetate were acquired from Mallinckrodt Baker (Phillipsburg, NY, USA). Silica gel 60 and chromatographic plates were obtained from Merck KGaA (Darmstadt, Germany). The infrared spectrum was recorded in solid phase using a Perkin-Elmer Paragon 1000 FT-IR spectrophotometer. NMR spectra were recorded at 298 K on a Bruker Avance DMX500 spectrometer operated at 500.13309 MHz for ^1^H-NMR and 125.77036 MHz for ^13^C-NMR. ^1^H- and ^13^C-NMR chemical shifts were reported relative to TMS and CDCl_3_, respectively. 

### 3.2. Plant Material

*Dodonaea viscosa* was collected at REBIOSH and a sample was deposited at the Herbarium of the Universidad Morelos (HUMO) with registration number: HUMO-26620. Vegetal identification was performed by Juan Carlos Juárez at the Centro de Investigación en Biodiversidad y Conservación (CIByC).

### 3.3. D. viscosa (DvDE) Dichloromethane Extract

The dried and ground material (1 kg) was extracted with dichloromethane (5 L) by maceration for 3 days/3 times. The solvent was eliminated under reduced pressure distillation with a Heidolph rotary evaporator. Dried extracts were compared by thin layer chromatography (TLC). Due to their chemical similarity, the extracts were mixed, giving a total yield of 2.03%.

### 3.4. HA Purification

DvDE extract (15.0 g) was fractionated in an open chromatographic column previously packed with 80 g of silica gel 60. An *n*-hexane-ethyl acetate gradient system was utilized as mobile phase, starting with 100% of the solvent of least polarity and finalizing with 100% ethyl acetate. This process was monitored by thin layer chromatography to afford a terpenoid mixture (F1). This fraction (8 g) was dissolved in a dichloromethane/acetone mixture (7:3, 20 mL) resulting in the crystallization of the major component that produced a blue spot in a thin layer chromatographic plate treated with Koumarosky reagent, indicating the presence of a terpene [23] and identified as *hautriwaic acid* (**1**): 1.5 g; colorless amorphous powder; m.p. = 175–176.5 °C; IR υ max = 2,921, 2,848, 1,652, 1,747, 870 cm^−1^; ^1^H-NMR (400 MHz, CDCl_3_): δ 7.35 (1H, m, H-15), 7.20 (1H, m, H16), 6.67 (1H, t, H-3), 6.25 (1H, m, H-14), 4.14 (1H, d, H-19), 3.68 (1H, d, H-19), 0.87 (3H, d, H-17), 0.76 (3H, s, H20). ^13^C-NMR (100 MHz, CDCl_3_), δ 172.13 (C18), 142.4 (C15), 141.1 (C-4), 138.0 (C-16), 138.0 (C-3), 124.9 (C-13), 110.5 (C14), 64.5 (C19), 45.9 (C-10), 41.5 (C-9), 38.3 (C-5), 38.2 (C-11), 35.8 (C-8), 30.79 (C-6), 26.3 (C-2), 26.2 (C-7), 18.1 (C-20), 17.8 (C-12), 16.5 (C-1), 15.3 (C17).

### 3.5. Animals

We used female CD-1 mice that weighed 25–30 g. Experiments were performed according to the Official Mexican Rule: NOM-062-ZOO-1999 Guidelines (Technical Specifications for the Production, Care, and Use of Laboratory Animals) and international ethical guidelines for the care and use of experimental animals. The experimental protocol was authorized by the Local Health Research Committee (IMSS, Register number: R-2010-1701-33). Mice were maintained at a temperature of 22 °C ± 3 °C, 70% ± 5% of humidity with 12-h light/dark cycles and food/water *ad libitum*. A control group received acetone as vehicle and indomethacin (Indo) was used as anti-inflammatory positive control.

### 3.6. Model of Acute Inflammation in Mice with TPA

Animal inflammation was induced following the method previously described by Payá [24]. Mice were grouped (eight individuals) and TPA (2.5 μg) dissolved in acetone (20 μL) was applied on the internal and external surface on the right ear to cause edema. Doses of 3 mg/ear of each treatment (DvDE extract, F1 and HA) were applied on the ear of each individual. For the isolated compound HA doses of 0.25, 0.5 and 1.0 mg/ear were used. Reference anti-inflammatory drug was administered at 1 mg/ear. All treatments were dissolved in acetone and applied topically on both ears immediately after the administration of TPA. Six hours after administration of the inflammatory agent, the animals were sacrificed by cervical dislocation. Circular sections of 6 mm in diameter were taken from both: the treated (t) and the non-treated (nt) ears, which were weighed to determine the inflammation. Percentage of inhibition was obtained using expression below:

Inhibition % = [Δw control − Δw treatment/Δw] [100]

where Δw = wt − wnt; wt is the weight of the section of the treated ear; wnt is the weight of the section of the non-treated ear.

### 3.7. Model of Chronic Inflammation in Mice with TPA

We employed the technique described by Lee [25]. Randomized groups of eight mice were formed. A solution with TPA (2.5 μg) dissolved in acetone (20 μL) was applied topically on alternate days (every 48 h) on the right internal and external auricular pavilion to cause inflammation. The studied substances, including DvDE extract (100 mg/kg) and HA (15 mg/kg), and Indo (5 mg/kg) were administered once daily (*i.p.*) for 10 days; the animals were sacrificed 6 h after the last day of treatment by cervical dislocation. Central section of 6 mm in diameter was taken from the right ear (with TPA) and from the left (vehicle); the sections were weighed to determine the percentage of inhibition obtained using the same expression:

Inhibition % = [Δw control − Δw treatment/Δw] [100]

### 3.8. Statistical Analysis

Results from delta weight (Δw = wt − wnt) are expressed as the mean ± Standard error of the mean (SEM) and these were analyzed using analysis of variance (ANOVA) and Bonferroni post-test (* *p* < 0.05).

## 4. Conclusions

The DvDE extract of *D. viscosa* at 3 mg/ear displays an important anti-inflammatory activity (97.8% inhibition of edema). Chromatographic purification of this extract provided a mixture of terpenes (**F1**) and a colorless crystalline compound that, according to IR and ^1^H-, ^13^C-NMR spectroscopic data, was identified as hautriwaic acid [17,18]. Both **F1** and this furoclerodane showed a potent anti-inflammatory effect on and acute model of TPA-induced edema with 96.9 and 87.1% inhibition at a dose of 1 mg/ear, respectively. This small increase of biological activity of **F1** respect to hautriwaic acid might be due to the fact that F1 may contain some other anti-inflammatory compounds. In the chronic inflammation model, hautriwaic acid displayed 64% of inhibition of edema at 15 mg/mg. HA showed an E_max_ = 97% of edema inhibition with an ED_50_ = 0.158 mg/ear. These inhibition values were similar or higher than those of the reference compound indomethacin when it was evaluated in the chronic test. Our study supports the use of *D. viscosa* as an anti-inflammatory treatment.
